# Cardiovascular risk associated with co-morbid insomnia and sleep apnoea (COMISA) in type 2 diabetics

**DOI:** 10.5935/1984-0063.20220018

**Published:** 2022

**Authors:** Matthieu Hein, Jean-Pol Lanquart, Anais Mungo, Gwenolé Loas

**Affiliations:** Erasme Hospital, Department of Psychiatry and Sleep Laboratory, Université libre de Bruxelles, ULB - Anderlecht - Bruxelles - Belgium.

**Keywords:** Sleep Apnea, Obstructive, Cardiovascular Diseases, Sleep Initiation and Maintenance Disorders, Diabetes Mellitus, Type 2, Polysomnography

## Abstract

**Objective:**

In the general population, co-morbid insomnia and sleep apnoea (COMISA) is associated with higher risk of cardiovascular diseases (CVD). However, despite a high prevalence of COMISA in type 2 diabetics, no study has investigated its potential implication in the negative cardiovascular outcome of this particular subpopulation. The aim of this study was therefore to examine the risk of CVD associated with COMISA in type 2 diabetics.

**Methods:**

Data from 471 type 2 diabetics recruited from the clinical database of the Erasme Hospital sleep laboratory were analysed. Only type 2 diabetics with SCORE index ≥5% were included in the group at high risk of CVD. Logistic regression analyses were conducted to examine the risk of CVD associated with COMISA in type 2 diabetics.

**Results:**

A high risk of CVD was present in 32.9% of type 2 diabetics. After adjustment for the main confounding factors associated with cardiovascular risk, multivariate logistic regression analysis revealed that unlike obstructive sleep apnoea syndrome or insomnia alone, only COMISA was associated with higher risk of CVD in type 2 diabetics.

**Discussion:**

In our study, we have demonstrated that unlike its components alone, only COMISA was associated with higher risk of CVD in type 2 diabetics, which highlights the importance of the central role played by the negative synergistic effect of COMISA on the cardiovascular outcome in this particular subpopulation. Thus, given these elements, more systematic research and adequate therapeutic management of COMISA seem to be necessary to allow better cardiovascular prevention in type 2 diabetics.

## INTRODUCTION

In the literature, there are several arguments for a special relationship between type 2 diabetes and obstructive sleep apnoea syndrome (OSAS). Indeed, the prevalence of type 2 diabetes is estimated at 30.1% in apnoeic individuals and that of OSAS may reach 54.5% in type 2 diabetics.^1,2^ In addition, type 2 diabetes is associated with a more frequent occurrence of OSAS whereas OSAS is a risk factor for the development of type 2 diabetes.^3,4^ Furthermore, the co-occurrence of OSAS in type 2 diabetes is a major public health problem since it is associated with higher cardiovascular morbidity and mortality.^5,6^ However, despite this major role played by OSAS in the risk of cardiovascular diseases (CVD) in type 2 diabetics, it has been shown that OSAS treatments had a limited impact on glycaemia control and cardiovascular risk factors in this particular subpopulation^7,8^ which seems to justify carrying out additional investigations to identify the potential cofactors involved in this particular relationship between OSAS and risk of CVD in type 2 diabetics.

In the general population, comorbid complaints of insomnia appear to play a major role in the particular relationship between OSAS and risk of CVD. Indeed, compared to OSAS alone, COMISA (co-morbid insomnia and sleep apnoea) is associated with more severe cardiovascular morbidity and mortality^9,10,11^ that may be explained by the existence of synergistic physiopathological mechanisms (deregulation of the hypothalamic-pituitary-adrenal axis, hyperactivation of the sympathetic nervous system and activation of proinfammatory mechanisms) between insomnia and OSAS.^12^ However, despite the significant prevalence of COMISA in type 2 diabetics,^13^ studies currently available in the literature have not investigated this potential negative synergic effect of COMISA on the risk of CVD in this particular subpopulation since they were limited to studying separately the cardiovascular impact of insomnia and OSAS.^14,15^ In addition, in studies assessing the cardiovascular impact of OSAS treatments in type 2 diabetics,^7,16,17^ the persistence of residual physiopathological mechanisms secondary to the lack to take into account this potential synergy between insomnia and OSAS could partially explain the limited effect of continuous positive airway pressure therapy on the risk of CVD in this subpopulation particular. Thus, in order to demonstrate this potential negative synergistic effect of COMISA on the risk of CVD in type 2 diabetics, it could be interesting to study the impact of COMISA on the cardiovascular outcome in this particular subpopulation.

Given the absence of reliable data available in the literature for healthcare professionals, the aim of our study was therefore to empirically investigate the potential negative synergic effect of COMISA on the risk of CVD in type 2 diabetics to allow the establishment of more effective cardiovascular prevention strategies in this particular subpopulation.

## MATERIAL AND METHODS

### Population

Based on the inclusion and exclusion criteria of this study, we recruited from the clinical database of the Erasme Hospital sleep laboratory 471 type 2 diabetics (354 men and 117 women) who performed a usual polysomnographic examination between January 01, 2002 and December 31, 2014. Only type 2 diabetics meeting our inclusion and exclusion criteria were therefore selected from the clinical database of the Erasme Hospital sleep laboratory to constitute the sample of type 2 diabetics included in this study.

These type 2 diabetics were referred to the sleep laboratory by physicians specialised in sleep medicine after an outpatient consultation during which a preliminary assessment of their complaints related to sleep, their ongoing psychotropic/ somatic treatments and their somatic/psychiatric comorbidities was systematically carried out in order to allow a frst diagnostic hypothesis. These polysomnographic examinations were performed in these type 2 diabetics to allow an objective assessment of their sleep complaints and exclude the presence of potential sleep disorders negatively impacting glycaemia control.

The inclusion criteria were age ≥40 years and the presence of type 2 diabetes meeting the diagnostic criteria of the *American Diabetes Association*.^18^ The exclusion criteria were the presence of diabetes other than type 2 diabetes (type 1 diabetes, gestational diabetes, latent autoimmune diabetes, maturity diabetes onset diabetes of the young, and secondary or iatrogenic diabetes), severe uncontrolled psychiatric pathology (psychotic or bipolar disorder), heavy uncontrolled somatic pathology (chronic hepatic pathology, chronic pancreatic pathology, severe cardiovascular pathology, chronic pulmonary pathology, severe renal pathology, autoimmune pathology and pathology altering the activity of the hypothalamic-pituitary-adrenal axis such as Cushing’s syndrome), infectious or infammatory pathology, ongoing pregnancy, central hypersomnia (narcolepsy or primary hypersomnia), parasomnia, predominantly central sleep apnoea syndrome, OSAS being treated before the sleep laboratory, current or past cranial trauma, current or past central nervous system lesions involving the respiratory centres, craniofacial or thoracic cavity malformations and current or past substance abuse.

### Method

#### Medical and psychiatric assessment of participants

Upon admission to the Erasme Hospital sleep laboratory, all individuals benefted from a review of their medical records and a complete somatic examination including blood test, electrocardiogram, day electroencephalogram, urinalysis and chest x-ray (only for subjects over 45 years old). These different steps allow a systematic diagnosis of potential somatic pathologies in individuals admitted to our unit.

Following this complete somatic assessment, type 2 diabetes was defned as present:

When one or more of the following criteria were fulfilled at admission: glycated haemoglobin (HbA1c) ≥6.5% or fasting plasma glucose ≥126mg/dl or two-hour plasma glucose ≥200 mg/dl during an oral glucose tolerance test or random plasma glucose ≥200 mg/dl in patients with classic symptoms of hyperglycaemia or hyperglycaemic crisis. In the absence of unequivocal hyperglycaemia, criteria 1–3 should be confrmed by repeat testing. In addition, diabetes must have begun in adulthood.^18^

Or when the patient at admission reported a biologically documented diagnosis of type 2 diabetes previously established by an outpatient diabetologist according to the diagnostic criteria of the *American Diabetes Association*.^18^

In addition, based on these criteria from the *American Diabetes Association*, type 2 diabetes was considered to be uncontrolled in case of glycated haemoglobin (HbA1c) ≥7.0% upon admission to the sleep laboratory or in case of diagnosis previously established by an outpatient diabetologist.^19^

Regarding the other cardiovascular risk factors, they were considered to be present on the basis of the following criteria:

Hypertension (diagnostic criteria of the *World Health Organization*): presence of mean systolic blood pressure ≥140 mmHg and/or mean diastolic blood pressure ≥90 mmHg or presence of self-reported diagnosis of hypertension previously established by an outpatient cardiologist or use of antihypertensive medication.^20^ In treated hypertensive individuals, controlled hypertension was defned as the presence of mean systolic blood pressure <140 mmHg and mean diastolic blood pressure <90 mmHg whereas uncontrolled hypertension was defned as the presence of mean systolic blood pressure ≥140 mmHg and/or mean diastolic blood pressure ≥90 mmHg.^20^

Dyslipidaemia (diagnostic criteria of the *International Diabetes Federation*): plasma triglyceride level ≥150 mg/dl or plasma HDL-cholesterol level <40 mg/dl for men or plasma HDL-cholesterol level <50 mg/dl for women or use of treatment for dyslipidaemia.^21^

Cardiovascular comorbidities (excluding hypertension): presence of one or more comorbid cardiovascular pathologies (cerebral vascular pathology, cardiac arrhythmias, coronary disease, heart disease, cardiac valve disease, peripheral vascular pathology and history of cardiac surgery).

Based on these different elements collected during this systematic somatic assessment, the SCORE index was calculated to determine the risk of CVD in type 2 diabetics included for this study. The SCORE index is used to estimate the risk of 10-year fatal cardiovascular event related to arteriosclerosis in individuals with age ≥40 years.^22,23^ The prediction model of the SCORE index integrates fve risk factors: age, sex, smoking status, systolic blood pressure and total cholesterol.^22,23^ Since diabetics were automatically considered as a subpopulation requiring intensive cardiovascular prevention during the development of the SCORE index, diabetes was not initially included in the risk factors used to calculate this index.^22,23^ However, the SCORE index may be used in diabetic populations after some adaptations: multiplication of SCORE index obtained by 4 for women and by 2 for men suffering from diabetes.^22,23^ Thus, after these recommended adaptations of SCORE index,^22,23^ the type 2 diabetics included in this study were considered to be at low risk of CVD in case of SCORE index <5% and at high risk of CVD in case of SCORE index ≥5%.

Then, after this somatic assessment, a complete psychiatric interview is systematically performed by a psychiatrist from our unit in all individuals admitted to the sleep laboratory in order to diagnose potential psychiatric pathologies according to the diagnostic criteria of the DSM-IV-TR.^24^ Thus, following this systematic psychiatric interview, the status (current or remitted) of potential major depressive episodes was determined according to the diagnostic criteria of the DSM-IV-TR.^24^

Finally, during their admission to the sleep laboratory, all these individuals completed a series of self-questionnaires to assess the severity of their subjective complaints of depression (BDI 13 items),^25^ daytime sleepiness (Epworth Sleepiness Scale [ESS])^26^ and insomnia (Insomnia Severity Index [ISI])^27^ (detailed description in previous study).^28^

#### Sleep evaluation and study

Upon admission to the sleep laboratory, all individuals benefted from a specific sleep interview to allow a complete assessment of their sleep-related complaints including sleeping habits, severity of self-reported insomnia complaints (difficulty falling asleep, repeated night-time awakenings, early morning awakening, and non-restorative sleep), symptoms of OSAS (snoring and self-reported apnoeas), symptoms of restless leg syndrome (RLS) (impatience of legs with or without abnormal sensations: aggravated by rest, partially or temporarily relieved by movements and increased during evening or night) and abnormal nocturnal movements (such as periodic limb movements during sleep [PLMs]).

Participants stayed in a sleep laboratory for two nights, including a frst night of habituation and a second night of polysomnography from which the data were collected for analysis. The patients went to bed between 22:00 - 24:00 and got up between 6:00 - 8:00, following their usual schedule. During bedtime hours, the subjects were recumbent and the lights were turned off. Daytime naps were not permitted.

The polysomnographic recordings performed in our unit meet the recommendations of the *American Academy of Sleep Medicine*.^29^ The applied polysomnography-montage was as follows: two electro-oculogram channels, three electroencephalogram channels (Fz-Ax, Cz-Ax and Oz-Ax, where Ax was a A1A2 mastoid reference), one submental electromyogram channel, electrocardiogram, thermistors to detect the oro-nasal airfow, fnger pulse-oximetry, a microphone to record breathing sounds and snoring, piezoelectric sensors to measure thoracic and abdominal breathing, and anterior tibialis electrodes. EEG montage (Fz-Ax, Cz-Ax and Oz-Ax) is a variant of EEG montage recommended by *American Academy of Sleep Medicine* (F4-A1, C4-A1 and O2-A1) that is used specifically in our sleep laboratory for clinical and scientific activity.^30,31^ Polysomnographic recordings were visually scored by specialised technicians according to the criteria of *American Academy of Sleep Medicine*.^32^

Obstructive apnoeas were scored if the decrease in air fow was ≥90% for at least 10 seconds whereas obstructive hypopnoeas were scored if the decrease in airfow was ≥30% for at least 10 seconds with a decrease in oxygen saturation of 3% or followed by microarousal.^33^ The obstructive apnoeahypopnoea index correspond to the total number of obstructive apnoeas and hypopnoeas divided by the period of sleep in hours. OSAS was considered to be present when the obstructive apnoea-hypopnoea index was ≥15/hour.^34^

PMLs were scored on the basis of the following strict criteria: 1) duration between 0.5 to 10 seconds, 2) interval between 5 and 90 seconds from leg movement onset and 3) movements had to be part of a series of ≥4 consecutive movements meeting these criteria.^35^ PLMs index corresponds to the total number of PLMs divided by period of sleep in hours. Moderate to severe PLMs were considered to be present when the PLMs index was ≥26/hour.^36^ Moreover, the diagnoses of RLS were made according to the diagnostic criteria of the *International Restless Legs Syndrome Study Group*.^37^

The diagnoses of insomnia disorder were performed according to the diagnostic criteria of the *American Academy of Sleep Medicine Work Group*^38^ whereas sleep deprivation was defned as sleep duration <6 hours.^39^

Thus, based on these different criteria, COMISA was considered to be present in case of co-occurrence between OSAS (obstructive apnoea-hypopnoea index ≥15/hour) and insomnia disorder.

### Statistical analyses

Statistical analyses were performed using Stata 14. The normal distribution of the data was verifed using histograms, boxplots, and quantile-quantile plots, and the equality of variances was checked using the Levene’s test.

In order to allow our analyses, we divided our sample of type 2 diabetics into a control group at low risk of CVD and a patient group at high risk of CVD. Only type 2 diabetics with SCORE index ≥5% were included in the patient group at high risk of CVD.

Since most continuous data followed an asymmetric distribution, we decided to use non-parametric tests for all these variables (Wilcoxon test) in order to highlight significant differences between the medians (P25-P75) observed in the different groups of type 2 diabetics. Finally, the categorical data were described by percentage and were analysed with Chi^2^ tests.

Univariate logistic regression models were used to study the risk of CVD associated with COMISA disorders (categorised: absent, sleep deprivation alone, insomnia alone, OSAS alone, COMISA) and the potential confounding factors (detailed description and references available in the Supplementary Data). In multivariate logistic regression models, the risk of CVD associated with COMISA disorders was only adjusted for significant confounding factors during univariate analysis. These different confounding factors were introduced hierarchically in the different multivariate logistic regression models.

The adequacy of the fnal model was verifed by the Hosmer and Lemeshow test whereas the specificity of the model was verifed by the Link test.

The results were considered significant when the p-value was <0.05.

## RESULTS

Compared to those with a low risk of CVD, type 2 diabetics with a high risk of CVD showed a reduction in sleep efficiency, total sleep time, % stage 2, % slow-wave sleep and % REM sleep as well as an increase in % wake after sleep onset, number of awakenings, micro-arousal index, obstructive apnoea-hypopnoea index, oxygen desaturation index, total time under 90% of Sao2 and PLMs index (Table 1). There were no significant differences between the two groups for sleep latency, sleep period time, % stage 1 and REM latency (Table 1).

**Table 1:** Polysomnographic data (n=471).

	Whole Sample (n=471)	Subjects with low cardiovascular risk (n=316)	Subjects with high cardiovascular risk (n=155)	P-value
**SL (min)**	23.3 (13.3 – 40.5)	23.0 (12.2 – 40.2)	25.0 (15.5 – 41.7)	0.115
**SE (%)**	76.1 (66.9 – 83.9)	79.3 (70.6 – 86.0)	71.0 (62.8 – 79.0)	<0.001
**SPT (min)**	445.5 (401.5 – 479.5)	442.8 (398.5 – 476.8)	451.0 (405.3 – 485.5)	0.281
**TST (min)**	368.0 (322.0 – 411.5)	378.5 (330.8 – 422.5)	350.7 (300.5 – 389.0)	<0.001
**% stage 1**	8.4 (5.4 – 11.8)	8.2 (5.4 – 11.0)	8.7 (5.5 – 13.4)	0.095
**% stage 2**	55.0 (47.8 – 62.6)	55.9 (50.1 – 63.9)	52.7 (43.7 – 60.8)	<0.001
**% SWS**	0.7 (0.0 – 3.7)	1.3 (0.0 – 5.1)	0.1 (0.0 – 1.8)	<0.001
**% REM sleep**	15.2 (10.5 – 19.2)	15.9 (11.3 – 19.8)	13.9 (8.3 – 17.6)	<0.001
**REM latency (min)**	85.7 (58.7 – 143.0)	84.0 (59.5 – 130.5)	91.5 (57.3 – 152.7)	0.258
**% WASO**	15.2 (8.6 – 23.9)	12.2 (7.4 – 21.3)	19.5 (14.1 – 29.4)	<0.001
**Number of awakenings**	36 (24 – 54)	34 (22 – 51)	42 (29 – 64)	<0.001
**Micro-arousal index**	10 (6 – 21)	9 (6 – 18)	12 (6 – 24)	0.046
**AHI**	8 (3 – 24)	7 (2 – 21)	15 (5 – 31)	<0.001
**ODI**	3 (1 – 11)	3 (1 – 8)	5 (2 – 18)	<0.001
**Total time under 90% of SaO2 (min)**	19.7 (1.7 – 102.0)	11.8 (0.6 – 77.4)	36.0 (7.3 – 137.5)	<0.001
**PLMs index**	24.4 (10.4 – 50.1)	23.6 (9.7 – 40.7)	30.6 (12.3 – 58.3)	0.018
	**Median (P25-P75)**	**Median (P25-P75)**	**Median (P25-P75)**	**Wilcoxon test**

AHI = apnoea-hypopnoea index, ODI = oxygen desaturation index, PLMs = periodic limb movements during sleep, REM = rapid eye movement, Sao2 = oxygen saturation, SE = sleep efficiency, SL = sleep latency, SPT = sleep period time, SWS = slow-wave sleep, TST = total sleep time, WASO = wake after sleep onset.

A high risk of CVD was present in 32.9% (n=155) of type 2 diabetics from our sample (Table 2). Aspirin therapy, antidiabetic therapy, overweight, obesity, alcohol consumption, dyslipidaemia status, hypertension status, cardiovascular comorbidities, depression status, sleep movement disorders and COMISA disorders were significantly associated with a high risk of CVD in type 2 diabetics (Table 2). In addition, type 2 diabetics with a high risk of CVD had higher age and lower BDI score than those with a low risk of CVD (Table 2). There were no significant differences between the two groups for gender, diabetes status, benzodiazepine receptor agonists, antidepressant therapy, caffeine consumption, smoking, snoring, ESS score, ISI score and CRP levels (Table 2). Finally, in type 2 diabetics with OSAS, COMISA was very frequent since the prevalence of comorbid insomnia disorders was 41.1% in this particular subpopulation (Table 2).

**Table 2:** Sample description (n=471).

Variables		Categories	%	Subjects with low cardiovascular risk	Subjects with high cardiovascular risk	P-value Chi^2^
Gender		Female (n=117)	24.8%	26.3%	21.9%	0.307
		Male (n=354)	75.2%	73.7%	78.1%	
Aspirin therapy		No (n=357)	75.8%	80.4%	66.5%	0.001
		Yes (n=114)	24.2%	19.6%	33.5%	
Antidiabetic therapy		Lifestyle changes (n=159)	33.7%	38.3%	24.5%	0.027
		Untreated (n=147)	31.2%	28.5%	36.8%	
		Oral medications (n=118)	25.1%	23.4%	28.4%	
		Insulin therapy (n=47)	10.0%	9.8%	10.3%	
Diabetes status		Controlled (n=289)	61.4%	68.2%	65.4%	0.532
		Uncontrolled (n=182)	38.6%	31.8%	34.6%	
Benzodiazepine		No (n=373)	79.2%	78.8%	80.0%	0.763
receptor agonists		Yes (n=98)	20.8%	21.2%	20.0%	
Antidepressant		No (n=351)	74.5%	75.0%	73.5%	0.734
therapy		Yes (n=120)	25.5%	25.0%	26.5%	
Caffeine		No (n=93)	19.7%	18.7%	21.9%	0.403
		Yes (n=378)	80.3%	81.3%	78.1%	
Smoking		No (n=382)	81.1%	82.0%	79.4%	0.497
		Yes (n=89)	18.9%	18.0%	20.6%	
Alcohol		No (n=311)	66.0%	69.6%	58.7%	0.019
		Yes (n=160)	34.0%	30.4%	41.3%	
Dyslipidaemia		No (n=127)	27.0%	29.1%	22.6%	0.023
		Without statin therapy (n=175)	37.1%	39.2%	32.9%	
		With statin therapy (n=169)	35.9%	31.7%	44.5%	
Hypertension		No (n=147)	31.2%	39.6%	14.2%	<0.001
		Untreated (n=68)	14.4%	13.3%	16.8%	
		Controlled (n=147)	32.2%	34.8%	23.9%	
Cardiovascular		No (n=362)	76.9%	79.8%	71.0%	0.034
comorbidities		Yes (n=109)	23.1%	20.2%	29.0%	
Depression		No (n=239)	50.7%	48.7%	54.8%	0.037
		Remitted (n=106)	22.5%	20.9%	25.8%	
		Current (n=126)	26.8%	30.4%	19.4%	
Snoring		No (n=81)	17.2%	15.8%	20.0%	0.259
		Yes (n=390)	82.8%	84.2%	80.0%	
Sleep movement		No (n=250)	53.1%	55.7%	47.7%	0.048
disorders		Moderate to severe PLMs (n=91)	19.3%	20.2%	17.4%	
		RLS alone or combined with PLMs (n=130)	27.6%	24.1%	34.9%	
COMISA		No (n=90)	19.1%	20.3%	16.8%	<0.001
		Sleep deprivation alone (n=56)	11.9%	10.1%	15.5%	
		Insomnia alone (n=150)	31.8%	38.9%	17.4%	
		OSAS alone (n=103)	21.9%	19.0%	27.7%	
		COMISA (n=72)	15.3%	11.7%	22.6%	
Cardiovascular risk		Low (n=316)	67.1%			
		High (n=155)	32.9%			
	**Median (P25-P75)**					**Wilcoxon test**
BMI (kg/m^2^)	30.3 (27.2 – 24.5)			30.1 (26.9 – 34.4)	30.4 (27.6 – 34.9)	0.287
				15.5%	5.8%	0.010^a^
		<25 (n=58)	12.3%	31.7%	36.8%	
		≥25 & <30 (n=157)	33.3%			
		≥30 (n=256)	54.4%	52.8%	57.4%	
Age (years)	55 (49 – 61)			51 (47 – 56)	64 (59 – 68)	<0.001
				67.1%	4.5%	<0.001^a^
		<55 (n=219)	46.5%	32.9%	95.5%	
		≥55 (n=252)	53.5%			
ESS	9 (6 – 13)			10 (6 – 14)	9 (5 – 12)	0.068
		≤10 (n=280)	59.4%	56.6%	65.2%	0.077a
		>10 (n=191)	40.6%	43.4%	34.8%	
BDI	4 (2 – 8)			5 (2 – 10)	4 (2 – 7)	0.031
ISI	14 (10 – 18)			15 (10 – 18)	13 (10 – 17)	0.123
CRP (mg/L)	2.1 (1.0 – 4.2)			2.3 (1.0 – 4.2)	2.1 (1.2 – 3.9)	0.903
		<1 (n=103)	21.9%	23.4%	18.7%	0.236^a^
		≥1 & <3 (n=194)	41.2%	38.6%	46.5%	
		≥3 (n=174)	36.9%	38.0%	34.8%	
						^a^ Chi^2^

BDI = beck depression inventory, BMI = body mass index, COMISA = co-morbid insomnia and sleep apnoea, CRP = C-Reactive Protein, ESS = Epworth sleepiness scale, ISI = insomnia severity index, OSAS = obstructive sleep apnoea syndrome, PLMs = periodic limb movements during sleep, RLS = restless legs syndrome.

COMISA, age ≥55 years, body mass index ≥25 kg/m^2^ & <30kg/m^2^, body mass index ≥30kg/m^2^, aspirin therapy, absence of antidiabetic therapy, use of oral hypoglycaemic medications, alcohol consumption, dyslipidaemia with statin therapy, untreated hypertension, controlled hypertension, uncontrolled hypertension, cardiovascular comorbidities, absence of depression and RLS alone or combined with moderate to severe PLMs were significantly associated with a high risk of CVD in our population of type 2 diabetics (Table 3).

**Table 3:** Univariate analysis (n=471).

Variables	Subjects with low cardiovascular risk	Subjects with high cardiovascular risk	OR (CI 95%)	P-value
Gender				
Female	70.9%	29.1%	1	0.307
Male	65.8%	34.2%	1.27 (0.80 to 2.00)	
Age (years)				
<55	96.8%	3.2%	1	<0.001
≥55	41.3%	58.7%	43.10 (19.49 to 95.31)	
BMI (kg/m^2^)				
<25	84.5%	15.5%	1	0.014
≥25 & <30	63.7%	36.3%	3.10 (1.42 to 6.78)	
≥30	65.2%	34.8%	2.90 (1.36 to 6.18)	
Aspirin therapy				
No	71.2%	28.8%	1	0.001
Yes	54.4%	45.6%	2.07 (1.34 to 3.19)	
Antidiabetic therapy				0.029
Lifestyle changes	76.1%	23.9%	1	
Untreated	61.2%	38.8%	2.02 (1.23 to 3.30)	
Oral medications	62.7%	37.3%	1.89 (1.12 to 3.19)	
Insulin therapy	66.0%	34.0%	1.64 (0.81 to 3.33)	
Diabetes status				0.532
Controlled	62.3%	37.7%	1	
Uncontrolled	59.4%	40.6%	1.13 (0.77 to 1.68)	
Benzodiazepine receptor agonists				
No	66.8%	33.2%	1	0.763
Yes	68.4%	31.6%	0.93 (0.58 to 1.50)	
Antidepressant therapy				
No	67.5%	32.5%	1	0.734
Yes	65.8%	34.2%	1.08 (0.70 to 1.67)	
Caffeine				
No	63.4%	36.6%	1	0.403
Yes	68.0%	32.0%	0.82 (0.51 to 1.31)	
Smoking				
No	67.8%	32.2%	1	0.497
Yes	64.0%	36.0%	1.18 (0.73 to 1.92)	
Alcohol				
No	70.7%	29.3%	1	0.019
Yes	60.0%	40.0%	1.61 (1.08 to 2.40)	
Dyslipidaemia				
No	72.4%	27.6%	1	
Without statin therapy	70.9%	29.1%	1.08 (0.65 to 1.80)	0.024
With statin therapy	59.2%	40.8%	1.81 (1.10 to 2.98)	
Hypertension				
No	85.0%	15.0%	1	
Untreated	61.8%	38.2%	3.52 (1.81 to 6.85)	<0.001
Controlled	74.8%	25.2%	1.91 (1.06 to 3.44)	
Uncontrolled	35.8%	64.2%	10.20 (5.60 to 18.56)	
Cardiovascular comorbidities				
No	69.6%	30.4%	1	0.035
Yes	58.7%	41.3%	1.61 (1.04 to 2.51)	
Depression				
No	64.4%	35.6%	1	
Remitted	62.3%	37.7%	1.10 (0.68 to 1.76)	0.039
Current	76.2%	23.8%	0.57 (0.35 to 0.92)	
Snoring				
No	70.4%	29.6%	1	0.260
Yes	68.2%	31.8%	0.75 (0.46 to 1.23)	
Sleep movement disorders				
No	70.4%	29.6%	1	
Moderate to severe PLMs	70.3%	29.7%	1.00 (0.59 to 1.70)	0.049
RLS alone or combined with PLMs	58.5%	41.5%	1.69 (1.09 to 2.63)	
COMISA				
No	71.1%	28.9%	1	
Sleep deprivation alone	57.1%	42.9%	1.85 (0.92 to 3.71)	
Insomnia alone	82.0%	18.0%	0.54 (0.29 to 1.01)	<0.001
OSAS alone	58.2%	41.8%	1.76 (0.97 to 3.22)	
COMISA	51.4%	48.6%	2.33 (1.22 to 4.46)	
CRP (mg/L)				
<1	71.8%	28.2%	1	
≥1 & <3	62.9%	37.1%	1.51 (0.90 to 2.53)	0.238
≥3	69.0%	31.0%	1.15 (0.67 to 1.96)	
ESS				
≤10	63.9%	36.1%	1	0.078
>10	71.7%	28.3%	0.70 (0.47 to 1.04)	

BMI = body mass index, COMISA = co-morbid insomnia and sleep apnoea, CRP = C-Reactive Protein, ESS = Epworth sleepiness scale, OSAS = obstructive sleep apnoea syndrome, RLS = restless legs syndrome, PLMs = periodic limb movements during sleep.

After adjustment for the main confounding factors associated with cardiovascular risk in the different models studied, multivariate logistic regression analysis revealed that unlike OSAS or insomnia alone, only COMISA was associated with higher risk of CVD in type 2 diabetics (Table 4).

**Table 4:** Multivariate analysis (n=471).

Variables	Model 1 OR adjusted (CI 95%)	P-value	Model 2 OR adjusted (CI 95%)	P-value	Model 3 OR adjusted (CI 95%)	P-value	Model 4 OR adjusted (CI 95%)	P-value
COMISA		<0.001		0.001		0.014		0.021
No	1		1		1		1	
Sleep deprivation alone	1.77 (0.87 to 3.61)		1.73 (0.79 to 3.80)		1.87 (0.67 to 5.20)		1.72 (0.61 to 4.84)	
Insomnia alone	0.56 (0.30 to 1.05)		0.61 (0.31 to 1.21)		0.71 (0.29 to 1.76)		0.85 (0.33 to 2.19)	
OSAS alone	1.68 (0.91 to 3.12)		1.75 (0.89 to 3.44)		1.55 (0.63 to 3.82)		1.58 (0.63 to 3.96)	
COMISA	2.26 (1.17 to 4.38)		2.40 (1.15 to 5.01)		3.41 (1.24 to 9.41)		4.00 (1.39 to 11.48)	

Model 1 = Model adjusted for aspirin therapy and antidiabetic therapy. Model 2 = Model adjusted for aspirin therapy, antidiabetic therapy, dyslipidaemia, hypertension and cardiovascular comorbidities. Model 3 = Model adjusted for aspirin therapy, antidiabetic therapy, dyslipidaemia, hypertension, cardiovascular comorbidities, age, BMI and alcohol. Model 4 = Model adjusted for aspirin therapy, antidiabetic therapy, dyslipidaemia, hypertension, cardiovascular comorbidities, age, BMI, alcohol, depression and sleep movement disorders. BMI = body mass index, COMISA = co-morbid insomnia and sleep apnoea, OSAS = obstructive sleep apnoea syndrome.

## DISCUSSION

In this study, we found that 32.9% of type 2 diabetics had a high risk of CVD. In addition, we have shown that unlike OSAS or insomnia alone, only COMISA was associated with higher risk of CVD in type 2 diabetics, which highlights the major role played by COMISA in cardiovascular risk for this particular subpopulation.

Following our results, we confrmed that type 2 diabetics are a subpopulation at high risk of CVD. Indeed, we have demonstrated that a high risk of 10-year fatal cardiovascular event was present in 32.9% of type 2 diabetics from our sample. Despite some methodological differences in recruitment, our results seem similar to those of the study by Nagpal and Bhartia (2008) where as in our study, the SCORE index showed a high risk of 10-year fatal cardiovascular event in 35.7% of type 2 diabetics.^40^ At the physiopathological level, the occurrence of several interrelated mechanisms (such as oxidative stress, pro-infammatory mechanisms, endothelial dysfunction, recurrent hypoglycaemia, autonomic neuropathies and synergistic effect of other cardiovascular risk factors [obesity, dyslipidaemia and hypertension]) could partially explain this particular relationship between type 2 diabetes and CVD.^41,42^ However, although there are arguments in favour of a reduction in these physiopathological mechanisms related to this deleterious cardiovascular outcome in case of intensive management of type 2 diabetes,^43^ CVD remain the leading cause of mortality in type 2 diabetics.^44^ These elements therefore indicate the need to identify the potential comorbidities involved in this less favourable cardiovascular prognosis in type 2 diabetics to allow better cardiovascular prevention in this particular subpopulation.

Consistent with the literature,^45,46^ we confrmed that OSAS is a frequent comorbidity (37.2%) in type 2 diabetics. In addition, we have shown that similar to the general population,^47^ comorbid complaints of insomnia are frequent in type 2 diabetics with OSAS. Indeed, in our study, comorbid complaints of insomnia were present in 41.1% of type 2 diabetics with OSAS, which highlights the importance of COMISA in this particular subpopulation. The existence of this frequent co-occurrence of OSAS and insomnia in type 2 diabetics could potentially be explained by the presence of some physiopathological mechanisms specific to this particular subpopulation favouring the concomitant development of these two sleep disorders.^48,49,50^ Indeed, the polyneuropathies induced by type 2 diabetes may promote the occurrence of obstructive respiratory events through alterations in central control of breathing and upper airway refexes whereas alterations in the hypothalamic-pituitary-adrenal axis induced by complex interactions between impaired glucocorticoid negative feedback sensitivity and some factors (such as hypoinsulinemia, hyperglycaemia and/or hypoleptinemia) may promote the development of insomnia in type 2 diabetics.^48,49,50^ Furthermore, we have demonstrated that compared to OSAS or insomnia alone, only COMISA was associated with higher risk of CVD in type 2 diabetics, which demonstrates the importance of the central role played by the negative synergistic effect of COMISA on the cardiovascular outcome in this particular subpopulation. At the physiopathological level, similar to the general population,^12,51^ this central role played by the negative synergy of COMISA on cardiovascular risk in type 2 diabetics could be explained by the presence of a cumulative effect of deleterious mechanisms related to OSAS and insomnia (deregulation of the hypothalamic-pituitary-adrenal axis, hyperactivation of the sympathetic nervous system and activation of pro-infammatory mechanisms) promoting the development of CVD. Thus, given these different elements, it seems to be necessary to systematically research for potential comorbid complaints of insomnia in type 2 diabetics with OSAS to allow the establishment of adapted therapeutic strategies and better cardiovascular outcome in this particular subpopulation.

At the therapeutic level, the highlight of this negative synergic effect of COMISA on the risk of CVD in type 2 diabetics could help better understand the limited impact of continuous positive airway pressure therapy alone on the cardiovascular risk in this particular population.^7,16,17^ Indeed, in type 2 diabetics with OSAS, the lack of adequate management of the potential comorbid complaints of insomnia could promote the maintenance of the pathophysiological mechanisms related to the development of CVD^52^ both by the direct negative impact of insomnia on the cardiovascular prognosis^53^ and by the indirect negative impact of insomnia on the compliance and efficacy of OSAS treatments.^54,55^ However, although the establishment of combined therapeutic strategies for COMISA could be a promising option to allow better cardiovascular prevention in some type 2 diabetics,^56^ these combined treatments should take into account the specific characteristics of this particular subpopulation in order to avoid the implementation of treatments with deleterious effects on the management of diabetes.^57^ Indeed, given the potential negative effect of most pharmacological treatments for insomnia on glycaemia metabolism and the few data available on the impact of therapeutic alternatives to continuous positive airway pressure therapy on glycaemia parameters in type 2 diabetics,^57,58,59^ the combination of cognitive-behavioural therapies for insomnia and continuous positive airway pressure therapy associated with lifestyle changes (weight loss and sports activity) seems to be the best option for frst-line treatment of COMISA in this particular subpopulation.^57^ Finally, regardless of this potential impact on the cardiovascular prognosis, the implementation of adequate treatments for insomnia and OSAS (even in the absence of cooccurrence) is essential given the negative impact of these two pathologies on life quality, diabetes self-care behaviours and patient-reported outcomes in type 2 diabetics.^60^

### Limitations

The results obtained in our study come from retrospective data that, even if they have been encoded in a systematic manner, cannot be verifed directly with the subject in most cases, which means that our results need to be replicated in prospective studies. Moreover, we focused only on type 2 diabetes, which means that our results cannot be generalized to other types of diabetes (such as type 1 diabetes, gestational diabetes, latent autoimmune diabetes, maturity diabetes onset diabetes of the young, and secondary or iatrogenic diabetes). Furthermore, although its use is validated in diabetic populations, the SCORE index was initially developed for the general population, which requires strict compliance with the adaptations recommended for diabetic populations in order to avoid the potential limitations related to its use in these particular subpopulations. Finally, our database only contains type 2 diabetics who have agreed to perform a sleep laboratory, which may also limit the generalisation of our results.

## CONCLUSIONS

In our study, we confrmed that type 2 diabetics are a subpopulation at high cardiovascular risk. In addition, we have demonstrated that unlike its components alone, only COMISA was associated with higher risk of CVD in type 2 diabetics, which highlights the importance of the central role played by the negative synergistic effect of COMISA on the cardiovascular outcome in this particular subpopulation. Thus, given these elements, more systematic research and adequate therapeutic management of COMISA seem to be necessary to allow better cardiovascular prevention in type 2 diabetics.

## SUPPLEMENTARY DATA Description of confounding factors included in univariate analyses

After a review of the literature on cardiovascular risk factors in type 2 diabetics^1,2,3,4,5,6,7,8,9^, the potential confounding factors included in this study were antidiabetic therapy (categorised: lifestyle changes, untreated, oral medications, insulin therapy), diabetes status (categorised: controlled, uncontrolled), dyslipidaemia (categorised: absent, without statin therapy, with statin therapy), hypertension (categorised: absent, untreated, controlled, uncontrolled), depression (categorised: absent, remitted, current), sleep movement disorders (categorised: absent, moderate to severe PLMs, RLS alone or combined with moderate to severe PLMs), body mass index (categorised: <25 kg/m^2^, ≥25 & <30 kg/m^2^, ≥30 kg/m^2^), age (categorised: <55 years, ≥55 years), CRP levels (categorised: <1 mg/L, ≥1 & <3 mg/L, ≥3 mg/L), ESS score (categorised: ≤10, >10) and as binary variables: gender, aspirin therapy, benzodiazepine receptor agonists, antidepressant therapy, alcohol consumption, smoking, caffeine consumption, cardiovascular comorbidities and snoring.
